# COVID-19: Perspectives for the management of dental care and education

**DOI:** 10.1590/1678-7757-2020-0358

**Published:** 2020-09-28

**Authors:** Bruno César de Vasconcelos Gurgel, Samuel Batista Borges, Raul Elton Araújo Borges, Patrícia dos Santos Calderon

**Affiliations:** 1 Universidade Federal do Rio Grande do Norte Centro de Ciências da Saúde Departamento de Odontologia NatalRio Grande do Norte Brasil Universidade Federal do Rio Grande do Norte, Centro de Ciências da Saúde, Departamento de Odontologia, Natal, Rio Grande do Norte, Brasil.

**Keywords:** Dental care, Dental education, Practice management, COVID-19, Infection control

## Abstract

The rapid and abrupt transmission pattern of the SARS-CoV-2 unleashed the current COVID-19 pandemic, as recognized by the World Health Organization in March 2020. Considering the high risk of transmission of the virus in dental environments and the specificities in clinical practice, COVID-19 posed immediate challenges for dental care and education. Due to the need to establish infection prevention and control guidance in dental health settings to enable a safe clinical practice, this review aims to list the challenges and perspectives in managing dental care in services and schools. This review employed materials collected from PubMed and the main guidelines and studies on the novel coronavirus to provide an overview of the clinical procedures and decisions made by health care personnel in dental offices and dental schools. We expect the COVID-19 scenario to promote significant changes in clinical practice and dental education; dentists should seek specific and particular regulations for dental practice established by their state or country. Biosafety checklists are strongly recommended for appointments at dental services and face-to-face activities in dental schools.

## Introduction

SARS-CoV-2 is a contagious RNA virus that causes the coronavirus disease 2019 (COVID-19) in the human population. This novel coronavirus is thought to spread primarily through respiratory droplets and fomites, but airborne transmission from person-to-person over long distances is unlikely.^3^ The virus can survive in aerosols for hours and on surfaces up to days.^4^^,^^5^ Pre-symptomatic or asymptomatic patients can also spread the virus, facilitating the transmission process.^4^^,^^6^^-^^8^

In December 2019, COVID-19 was first diagnosed in a human and recognized as a serious respiratory disease in Wuhan city, Hubei province, in China. Since then, a huge scientific task force has been assembled to collect information about the disease.^9^ Several studies have been developed to identify its biological mechanism, infectious agent, contagion and transmission modes, diagnostic tests, and to search drugs and vaccines to tackle the virus.

Due to the rapid and abrupt worldwide transmission pattern of the COVID-19, in March 2020 the World Health Organization declared the outbreak a pandemic.^10^ Despite global efforts to stem the spread of the disease, the incidence rate of COVID-19 is continuously increasing: the latest issue reported over 29,155,581 laboratory-confirmed cases and more than 926,544 deaths worldwide.^11^

During this novel coronavirus pandemic, several countries have adopted restrictive measures and actions to decrease close contact among people, for preventing the spread of the virus and limiting contagion (social isolation and self-quarantine). Such measures pose immediate challenges for dental health care personnel (DHCP) and dental education institutions of all affected regions.

### Current Scenario

A Chinese team of scientists quickly isolated the virus from patients and sequenced its genome (29,903 nucleotides). They verified that although SARS-CoV-2 is different from SARS-CoV – which causes the Severe Acute Respiratory Syndrome (SARS) identified in 2003 in China – they share the same host receptor, human angiotensin-converting enzyme 2 (ACE2).^12^ In other words, SARS-CoV-2 can effectively use ACE2 as a receptor to invade humans cells, facilitating transmission.^4^

ACE2 receptors on epithelial cells of the oral mucosa and on gland ducts showed to be early targets of SARS-CoV infection^13^ and possibly of SARS-CoV-2. This, along with other evidence, highlights the hidden capability of saliva for the early detection of various viral, bacterial, or systemic diseases (Epstein-Barr virus - EBV; Cytomegaloviruses - CMV; Varicella-Zoster virus - VZV, Human papillomavirus - HPV).^14^

Scientists isolated the novel coronavirus from COVID-19 patients saliva, which could become a major source of viral load.^15^ Considering that, both DHCP and patients are at infection risk by routine dental practices that enable contact with mucous membranes, microorganisms, large drops of water, saliva, blood (among other body fluids), and with debris thrown into the air due to the use of air-water syringes, rotatory instruments, or ultrasonic scalers,.^4^^,^^16^ The high ACE2 expression in salivary glands makes this protein a potential target for COVID-19.^17^ Moreover, SARS-CoV RNA can be reportedly detected in saliva before lung lesions appear,^18^ indicating that asymptomatic COVID-19 spread may derive from infected saliva.^17^

A recent study proved the consistent presence of SARS-CoV-2 in the saliva of 11 patients admitted for COVID-19 infection from the first day of hospitalization in a Hong Kong hospital.^15^ Material was collected by instructing patients to cough saliva from their throat into a sterile container and referred to laboratory analysis. This study results demonstrate the potential use of saliva in developing more convenient tests and in early detecting COVID-19.^14^ When compared to the currently recommended respiratory samples, such as the nasopharyngeal and oropharyngeal smears,^19^ collecting saliva is technically easier and more comfortable to the patient, so it should be explored in the current pandemic scenario. Using saliva to diagnose COVID-19 is safer for health care providers and could expand testing capacities worldwide.

Telemedicine has granted electronic consultations even more visibility during this COVID-19 pandemic.^20^ Such approach allows patients screening and avoids spreading the virus among patients, health professionals, and the community.^21^ Although recent evidence support the adoption of teledentistry,^22^^,^^23^ conclusive study outcomes are not sufficient to evaluate the efficacy, cost-effectiveness, and long-term use of this approach and support evidence-based policy decisions^23^ in private or public services.

Teledentristy is feasible for some areas in dentistry in providing remote consultations for developing treatment plans, promoting preventive care, and supervising home procedures such as pediatric dentistry, oral medicine, orthodontics, prosthodontics, periodontics, and restorative dentistry. A recent systematic review found telediagnosis and teleconsultation to be the most common applications, grounded on store-and-forward technology and real-time encounters.^23^ A future perspective is improving and developing technology to provide access to teledentistry and extend the service in several countries.

A study published on April 19, 2020 reveals the progress and challenges in developing drugs to tackle COVID-19, with some updates.^9^ Although no specific drug is currently suitable for treating COVID-19, this study lists some medication and alternative treatments, such as favilair, remdesivir, chloroquine, and hydroxychloroquine.^9^ In an attempt of developing a method to confer acquired immunity against the disease, several SARS-CoV-2 vaccine prototypes have been developed and tested by countries around the world. Canada, China, United Kingdom, Russia, Brazil, and United States have been working to develop a vaccine in a short time. It is estimated that it will take at least one year, after pre-clinical and clinical studies, to make vaccine available to the population.^9^ Producing and testing these products will demand high costs for the governments.

According to Alharbi, Alharbi, and Alqaidi^24^ (2020), suspending dental practices during the course of the novel coronavirus pandemic may contribute to reducing infection, but will increase the suffering of individuals requiring emergency dental care.^24^ Such measure may also contribute in overcrowding hospital emergency departments, evincing great importance in providing dental care, even during a global pandemic. Some countries are gradually reopening their economic activities, including dental practices. In this scenario, it is necessary to establish infection prevention and control guidance in dental health settings to enable a safe clinical practice (both in dental offices and schools), during and after the pandemic.

Dental education is being dramatically impacted due to the higher risk of contamination of patients, teaching staff, students, and DHCP, as these individuals circulate in an environment that favors virus spreading. As an example, the number of cases among health care professionals (especially physicians), nurses, and dentists is high.^4^^,^^9^^,^^16^ This review aims to list the challenges and perspectives in managing dental care and education during and after the course of the COVID-19 pandemic.

This is not a systematic review. This manuscript was written based on material collected from the PubMed database, using the search terms: “coronavirus”, “SARS-CoV-2”, “COVID-19”, “dentistry”, “dental”, “oral cavity”, “biofilm”, “saliva”, “dental public health”, “infection control”, “prevention”, “education”, and “practice management;” the search was limited to texts written in English.

### Infection prevention and control for dental practice

Dental professionals should be prepared to identify patients with COVID-19 and to take extra-protective measures required during clinical practice to prevent the transmission.^4^ We will present infection prevention and control measures indicated for these professionals before, during, and after dental procedures implicating a possible droplet and aerosol transmission of COVID-19, most important concerns in dental offices. The presented recommendations are based on the guidance for healthcare workers,^19^ for infection prevention and control during healthcare when COVID-19 is suspected,^25^ and the American Dental Association (ADA) interim guidance for minimizing risk of COVID-19 transmission.^26^

The ADA proposed three algorithms serving as interim guidance to assist dentists and dental offices in making informed decisions concerning patient triage, evaluation, and treatment during the COVID-19 pandemic: (1) triaging patients for emergency and urgent dental care, (2) screening to identify COVID-19 infection in emergency and urgent dental patients, to determine whether patients can be treated at the dental office (3) and assessing patients' risk during the pandemic.^27^ The guidance also recommends dental offices and schools to take actions for preventing and controlling the infection.

### Before the dental procedure

Initially, dental health care personnel (DHCP) must prepare the dental team.^26^ DHCP should have received influenza vaccination and professionals presenting influenza-like illness (ILI) (fever, cough, sore throat, muscle pain) should avoid showing up to work. All DHCP must self-monitor by observing possible respiratory symptoms (cough, shortness of breath, sore throat) and check their temperatures twice a day. Officers must control the available personal protective equipment (PPE - surgical masks, surgical gowns, surgical gloves, face shields) supplies. Appointments should be scheduled far enough apart to minimize possible contact with other patients in the waiting room. Patients should be requested not to take companions to their appointments, except when they require assistance (pediatric patients, people with special needs, older adults).

Then, personnel must screen patients for COVID-19 status and triage them for dental treatment, providing early recognition and source control (isolating patients with suspected COVID-19).^26^^,^^28^ Patient's interview should preferably be conducted by telephone, text and images messaging mobile applications, or video conference, which can be useful with instant communication and quick decisions before the visit.^21^^,^^23^ The ADA also recommends dentists to apply a screening questionnaire with appropriate questions to track suspected COVID-19 patients. Questions may address whether, in the last 14 days, the patient has been in close contact with people diagnosed or under investigation for COVID-19 or has had fever, sore throat, cough, or shortness of breath. Screening should be performed at appointment confirmation, 24 hours before, or when the patient presents for treatment.^26^

Public areas and clinical environments must be properly and rigorously cleaned and disinfected. Thoroughly cleaning environmental surfaces with water and detergent and applying high-level disinfectants (such as 3% sodium hypochlorite) are effective and sufficient procedures.^25^ Medical devices/equipment and utensils, including knobs, chairs and tables, should be cleaned frequently following safe routine procedures.

Upon patient arrival, the waiting room should allow a social distance of at least 2 meters between people.^27^ Consultations must be scheduled by appointment to avoid overcrowding. The service must provide supplies to maintain hygiene/breathing level by offering a medical mask to patients while they wait and asking them to perform hand hygiene (cleaning hands with 70% alcohol for 20 to 30 s, or with soap and water from 40 to 60 s).^25^

Professionals should perform another screening to confirm patients' health condition by checking their temperature, preferably using a non-contact forehead thermometer or infrared cameras. If the patient exhibits a temperature higher than 37.5°C or signs of respiratory diseases, dental care must be postponed for 14 days and patients must follow the recommendations previously listed.^29^^,^^30^

No clinical study supported using mouthrinse before dental procedures, nor any specific mouthrinse virucidal efficacy against SARS-CoV-2.^31^ Although chlorhexidine is considered the gold standard for oral antiseptics, according to the Chinese Clinical Guidelines for COVID-19 pneumonia diagnosis and treatment (5th edition) published by the National Health Commission of the People's Republic of China, it may not be effective in killing SARS-CoV-2.^4^^,^^31^ As SARS-CoV-2 may be vulnerable to oxidation, the ADA suggests using mouthrinse with 1.5% hydrogen peroxide or 0.2% povidone prior to conducting procedures,^26^ as well as 0.05% to 0.1% cetylpyridinium chloride, which seems to provide additional protection against upper respiratory infections.^32^

### During the dental procedure

During dental procedures, standard precautions include preparing all instruments, performing hand/respiratory hygiene, using adequate PPE and disposable covers for total protection.^8^ According to Peng, et al.^4^ (2020, adapted according to WHO^8^ guidelines), three-level protective measures of DHCP are recommended for specific situations:^4^ (1) *Primary protection* (standard protection for staff in clinical settings), which consists of wearing disposable working cap, disposable surgical mask, working coat, protective goggles or face shield, and disposable latex or nitrile gloves; (2) *Secondary protection* (advanced protection for dental professionals), wearing a disposable doctor cap and surgical mask, protective goggles, face shield, and working clothes with disposable isolation clothing, and disposable latex gloves; and (3) *Tertiary protection* (strengthened protection for contact with suspected or confirmed COVID-19 patient), all secondary-protection level added to disposable N95 masks and impermeable shoe covers.

Dental care should be fast and as minimally invasive as possible, avoiding aerosol-generating procedures. Dental care environment should employ high-efficiency particulate arresting (HEPA) air purifiers to ensure DHCP and patients safety (Chen and Zhao, 2020). For biosafety control, all filters must be replaced and disposed of in regular intervals.^33^ To minimize the risk of cross-infection, dentists should use single-use devices, such as disposable air/water syringe tips, disposable handpieces, and plastic wraps for all exposed surfaces^27^. Services should limit complementary exams, as intraoral images, and perform extraoral radiographs to avoid increased salivary flow rate and pharyngeal reflex, often caused during intraoral imaging.^4^^,^^24^

Whenever possible, procedures should employ a rubber dam, to reduce aerosols and microorganisms spread,^26^^,^^27^^,^^34^ and a four-handed operation, regardless of the rubber dam. Extra high-volume suction devices should be used in conjunction with regular suction for aerosols, fomites and saliva.^4^^,^^27^^,^^34^ When rubber dam use is unfeasible, manual devices for removing dental cavities and performing periodontal treatment are recommended to minimize aerosol production as much as possible.^4^^,^^27^ During procedures, patients should wear a protective coat, disposable working cap, and protective goggles.

Patients with suspected or confirmed COVID-19 must be attended only for emergency treatment and instructed to wear a disposable surgical mask. Appointments should be scheduled far enough apart to avoid possible contact with other individuals. Treatment should be preferably performed in a negative pressure/air borne precaution room.^29^

### After the dental procedure

According to the ADA, after dental appointment completion, professionals should reinforce instructions on social distance and infection control etiquette (hand and respiratory hygiene).^26^ Special attention must be paid to cleaning and disinfecting dental office, reusable PPE (goggles and facial shields), and non-disposable and non-sterilizable equipment (dental x-ray equipment, dental chair and light). Other instruments that go into direct contact with fluids and the oral cavity must be sterilized in an autoclave. Oral hygiene care should be reinforced during this period.

### Professionals with symptoms or suspected COVID-19

Professionals exhibiting symptoms or those suspected of COVID-19 (after being exposed to a confirmed COVID-19 patient) must discontinue all healthcare interactions for a 14-day period,^19^ tested for COVID-19, and quarantined for 14 days.^25^ If test result comes out positive, patients attended in the previous week and DCHP working with the professional must be notified, instructed to self-quarantine at home for 7 days, and report any fever or influenza-like illness experience to local health services.

### Dental education

Worldwide, most dental schools suspended teaching activity due to pandemic spread to reduce virus transmission and the spread of COVID-19 in dental departments. As a consequence, this strategy has exerted numerous effects on dental education, such as: interrupting face-to-face instruction, including pre-clinical and clinical activities; interrupting patients' assistance; discontinuing development of human and some animal studies, especially in postgraduate programs; cancelling scientific congresses and conferences; abruptly changing academic calendars, including extra semesters; and postponing graduation ceremonies and new students entry.^35^^,^^36^ We should also consider the negative impact on the mental health of professionals, professors, and students.^36^^,^^37^^,^^38^

Considering that, a major concern among dental schools is restarting activities. As dental schools cannot extensively adopt web-based teaching for practicing, some urgent considerations are necessary.^38^^,^^39^^,^^40^ Resuming dental schools activities in classroom and clinics depends on the course of this pandemic in each country and specific field. Currently, some dental schools have resumed or prepared to reopen after government decisions,^40^ what urges for some recommendations on how to organize dental education considering the “new normal” in Dentistry.

### Education

The pandemic forced many dental schools to temporarily adapt to a fully-virtual curriculum. To accommodate the lasting effects of COVID-19, some strategies adopted for distance education should be maintained in a hybrid-teaching format in the upcoming months.^35^^,^^38^ Considering that, institutions should use remote strategies, such as free digital platforms and software – mediated synchronously (live) and asynchronously (recordings),^37^^,^^41^ – videoconferences, online forums and lectures, critical review of scientific articles, clinical cases discussion, problem-based learning tutorials, and demonstrations of laboratory and clinical techniques, to avoid unnecessary agglomerations.^37^^,^^38^^,^^41^^,^^42^ These technology-mediated pedagogical approaches can help creating an active learning environment and cultivate positive attitudes towards the teaching-learning process among dentistry students.^39^^,^^41^^,^^42^

### Preclinical practice

Schools should also encourage preclinical activities in the simulation laboratory using mannequins, given the possible reduced availability of clinical practice with patients, especially for first-year students of graduation courses.^35^^,^^37^ Another alternative is the use of digital strategies, by software capable of creating and reproducing virtual three-dimensional models that can be accessed by computers and mobile devices. Such approaches may enable students to learn techniques and concepts applied into different dental specialties.^43^^,^^44^^,^^45^ However, remote strategies should not replace clinical practice with patients in acquiring abilities inherent to the dental training.

### Clinical practice

As dental school environment aggravates agglomeration, some prevention strategies should be implemented to reduce contamination among those individuals before dental treatment (at-home patients and those entering the dental school). Patients should undergo screening, to investigate their health conditions and suspected cases of COVID-19,^42^ by telephone, 24 hours before consultation, or at first-contact for the appointment, as soon as the patient arrives at the dental school. Patients with difficulty in explaining their oral problem by telephone must receive consultation by video. Those suspected of COVID-19 must be referred to another local health service.^42^

When patients arrive at the dental school, practitioners must register their body temperature and apply a current health status questionnaire to avoid cross-infection during appointment.^19^ Patients presenting risk factors (advanced age and other comorbidities) should have their appointments postponed as far as possible, or be treated in a remote area.

Visible recommendations should be available outside the dental school, instructing patients on hygiene and respiratory etiquette. The school is recommended to strictly control the number of patients in the waiting room and display chairs within a 2-meter distance. Patients should only take companions into the clinic if necessary,^16^ and are advised to leave all belongings outside.

Students should provide all necessary materials before the dental procedure to limit contact with other students and personnel and to perform the procedure as fast as possible. Special care must be taken with shared dental consumables. Since procedures become more complex as the graduation course advances, dental students generally take longer to perform procedures, especially at clinical-stage, in the beginning of the course. The risk of cross-infection is higher at this stage because students spend more time in contact with the patient, depending on the nature of the procedure to be performed.

Four-hand work is extremely necessary during dental procedures to prevent contagion and speed up the appointment. Hand hygiene should be rigorously performed at each appointment and PPE should be available for all dental practitioners. Before dental treatment, prescribing mouthrinses is recommended – rinses should contain 1% hydrogen peroxide, or 0.2% to 1% povidone, or 0.05% to 0.1% cetylpyridinium chloride agents, and are particularly indicated when a rubber dam is not used.^16^^,^^32^ Students should opt for procedures that produce no or little aerosol and for using manual instruments when possible. Patients must wear a protective coat, disposable working cap, and protective goggles during procedures.

After completion, all potentially contaminated surfaces should be cleaned. Sterilizable instruments should be disinfected using enzymatic detergents, which is effective in removing organic matter and rapidly decomposing adhered blood and body fluids. Other dental settings should be cleaned with 70% alcohol. Surfaces should be disinfected with 0.1% sodium hypochlorite and 70% alcohol.^16^

After each practical assistance, disposable PPE should be discarded and the rest of the equipment disinfected. As the virus remains alive in droplets suspended in the air, PPE should not be removed before exiting the area,^16^ and during removal touching any external part of the equipment must be avoided, including disposable masks and caps. For a safer practice, the dental team should be constantly monitored by rapid diagnostic tests for COVID-19.^39^

Regarding infection control measures, Emami^36^ (2020) argues that dental teams are familiar in treating patients with infectious diseases (hepatitis, HIV) and routinely working with strict environmental control and PPE. As an example, a recent multinational study approaching dentists' knowledge, attitudes, and practices regarding COVID-19 revealed that 92.7% of the participants presented high/good knowledge and 79.5% high/good care practices, evincing these professionals promising role in controlling COVID-19 infections.^46^ Choi and Kim^47^ (2016) investigated factors influencing the prevention of the MERS-CoV by applying a questionnaire to nursing students, and found that attitude was the most affected preventive behavior. Perceived risk, older students, education level during graduation course, and female respondents were also associated with a higher perception.

Park, et al.^48^ (2016) reported a medical school experience during a Middle East Respiratory Syndrome (MERS) outbreak and suggested schools to consider distant education with online technology, distant lectures, and tutorials. Key decisions to avoid student infection were based on (1) student safety, (2) minimizing learning loss, and (3) reducing students and staff anxiety and concern. The medical school assessed ongoing risk and developed contingency plans to balance staff and student safety and created a monitoring team. It also adopted precautions, such as repeated hand washing and sanitizing, taking temperatures twice a day, and wearing masks. Bulletin boards, emails, and text messages included reminders and recommended precautions in case of initial symptoms of MERS.^48^

Dental schools will eventually reopen according to government schedules. However, school staff must develop an strategic plan to avoid any learning loss from students and reduce the risk of COVID-19 infection among patients, lecturers, students, and other staff. Adopting such strategies in the daily life of dental schools will strengthen dental education now and in future unexpected crisis.

### Perspectives for Future Research

The COVID-19 pandemic has urged scientific researchers and nanotechnology experts to study and develop devices capable of performing diagnosis and therapy. Mesenchymal stem cells (MSCs) showed good results in the treatment of patients with COVID-19 pneumonia, probably by preventing or reversing pro-inflammatory cytokines overactivation, allowing tissue repair and maintaining mortality as low as possible.^49^^,^^50^^,^^51^ Widely addressed in regenerative medicine and dentistry, MSCs demonstrated a valuable potential clinical application due to their repairing behavior and immunomodulatory properties.^52^^,^^53^ In this context, Dentistry is outlined for developing future research and therapies involving MSCs, as sources are easily accessible and of low biological cost and risk.^53^

The theranostic approach^54^ could also be advanced to manage some diseases using nanomaterials – that is, as a drugs carriers for specific action in tissues or cells using nanobiotechnology and translational research. Due to its predictability, preventive, personal, and participatory potential,^54^^,^^55^ theranostics promises to increase clinical performance (including in Dentistry), reducing costs and helping identifying the ideal and individualized treatment for each patient, at the right moment, in an analysis beyond the current pandemic scenario.

## Conclusion

Dental professionals must be prepared to face any imminent challenge in clinical practice imposed by infectious diseases, e.g. COVID-19, and define a conduct for patient care, by postponing consultations and referring individuals to quarantine at home or to healthcare centers and hospitals. Dentist role in preventing and monitoring viral infections should be constantly reviewed. Following prevention guidelines proposed by the WHO is important to prevent SARS-CoV-2 action in the population until drugs and vaccines are developed.

Dentists should seek specific regulations for dental care established by their state or country regarding the COVID-19 pandemic. Biosafety checklists should also be consulted for managing protective personal equipment, dental settings and devices, and surrounding spaces – as those used before, during, and after dental appointments. As significant changes are expected in clinical practice and dental schools, individuals should seek further and rapid information to handle and improve this issue.

## References

[1] 1- Lu R, Zhao X, Li J, Niu P, Yang B, Wu H, et al. Genomic characterisation and epidemiology of 2019 novel coronavirus: implications for virus origins and receptor binding. Lancet. 2020;395(10224):565-74. doi: 10.1016/S0140-6736(20)30251-8 10.1016/S0140-6736(20)30251-8 PMC7159086 32007145

[2] 2- Mahase E. China coronavirus: WHO declares international emergency as death toll exceeds 200. BMJ. 2020;31;368:m408. doi: 10.1136/bmj.m408 10.1136/bmj.m408 32005727

[3] 3- Holshue ML, DeBolt C, Lindquist S, Lofy KH, Wiesman J, Bruce H, et al. First case of 2019 novel coronavirus in the United States. N Engl J Med. 2020;382(10):929-36. doi: 10.1056/NEJMoa2001191 10.1056/NEJMoa2001191 PMC7092802 32004427

[4] 4- Peng X, Xu X, Li Y, Cheng L, Zhou X, Ren B. Transmission routes of 2019-nCoV and controls in dental practice. Int J Oral Sci. 2020;12(1):9. doi: 10.1038/s41368-020-0075-9. 10.1038/s41368-020-0075-9 PMC7054527 32127517

[5] 5- van Doremalen N, Bushmaker T, Morris DH, Holbrook MG, Gamble A, Williamson BN, et al. Aerosol and surface stability of SARS-CoV-2 as compared with SARS-CoV-1. N Engl J Med. 2020;382(16):1564-67. doi: 10.1056/NEJMc2004973 10.1056/NEJMc2004973 PMC7121658 32182409

[6] 6- Bai Y, Yao L, Wei T, Tian F, Jin DY, Chen L, et al. Presumed asymptomatic carrier transmission of COVID-19. JAMA. 2020;323(14):1406-7. doi: 10.1001/jama.2020.2565 10.1001/jama.2020.2565 PMC7042844 32083643

[7] 7- Lai CC, Liu YH, Wang CY, Wang YH, Hsueh SC, Yen MY, et al. Asymptomatic carrier state, acute respiratory disease, and pneumonia due to severe acute respiratory syndrome coronavirus 2 (SARS-CoV-2): facts and myths. J Microbiol Immunol Infect. 2020;53(3):404-12. doi: 10.1016/j.jmii.2020.02.012 10.1016/j.jmii.2020.02.012 PMC7128959 32173241

[8] 8- World Health Organization. Infection prevention and control during health care when COVID19 is suspected [Internet]. Geneva: WHO; 2020 [cited 2020 Apr 21]. Available from: https://apps.who.int/iris/rest/bitstreams/1272420/retrieve

[9] 9- El-Aziz TM, Stockand JD. Recent progress and challenges in drug development against COVID-19 coronavirus (SARS-CoV-2): an update on the status. Infect Genet Evol. 2020;83:104327. doi: 10.1016/j.meegid.2020.104327. 10.1016/j.meegid.2020.104327 PMC7166307 32320825

[10] 10- World Health Organization. Virtual press conference on COVID-19 – 11 March 2020 [Internet]. Geneva: WHO; 2020 [cited 2020 Apr 21]. Available from: https://www.who.int/docs/default-source/coronaviruse/transcripts/who-audio-emergencies-coronavirus-press-conference-full-and-final-11mar2020.pdf

[11] 11- World Health Organization. Coronavirus disease (COVID-19) dashboard [Internet]. Geneva: WHO; 2020 [cited 2020 July 13]. Available from: https://covid19.who.int/

[12] 12- de Wit E, van Doremalen N, Falzarano D, Munster VJ. SARS and MERS: recent insights into emerging coronaviruses. Nat Rev Microbiol. 2016;14(8):523-34. doi: 10.1038/nrmicro.2016.81 10.1038/nrmicro.2016.81 PMC7097822 27344959

[13] 13- Liu L, Wei Q, Alvarez X, Wang H, Du Y, Zhu H, et al. Epithelial cells lining salivary gland ducts are early target cells of severe acute respiratory syndrome coronavirus infection in the upper respiratory tracts of rhesus macaques. J Virol. 2011;85(8):4025-30. doi: 10.1128/JVI.02292-10 10.1128/JVI.02292-10 PMC3126125 21289121

[14] 14- Khurshid Z, Zafar MS, Khan RS, Najeeb S, Slowey PD, Rehman IU. Role of salivary biomarkers in oral cancer detection. Adv Clin Chem. 2018;86:23-70. doi: 10.1016/bs.acc.2018.05.002 10.1016/bs.acc.2018.05.002 30144841

[15] 15- To KK, Tsang OT, Chik-Yan Yip C, Chan KH, Wu TC, Chan JM, et al. Consistent detection of 2019 novel coronavirus in saliva. Clin Infect Dis. 2020;71(15):841-3. doi: 10.1093/cid/ciaa149 10.1093/cid/ciaa149 PMC7108139 32047895

[16] 16- Izzetti R, Nisi M, Gabriele M, Graziani F. COVID-19 Transmission in dental practice: brief review of preventive measures in Italy. J Dent Res. 2020;99(9):1030-8. doi: 10.1177/0022034520920580 10.1177/0022034520920580 32302257

[17] 17- Xu J, Li Y, Gan F, Du Y, Yao Y. Salivary glands: potential reservoirs for covid-19 asymptomatic infection. J Dent Res. 2020;99(8):989. doi: 10.1177/0022034520918518 10.1177/0022034520918518 32271653

[18] 18- Wang WK, Chen SY, Liu IJ, Chen YC, Chen HL, Yang CF, et al. Detection of SARS-associated coronavirus in throat wash and saliva in early diagnosis. Emerg Infect Dis. 2004;10(7):1213-9. doi: 10.3201/eid1007.031113 10.3201/eid1007.031113 PMC3323313 15324540

[19] 19- World Health Organization. Risk assessment and management of exposure of health care workers in the context of COVID-19 [Internet]. Geneva: WHO; 2020 [cited 2020 Apr 21]. Available from: https://apps.who.int/iris/bitstream/handle/10665/331496/WHO-2019-nCov-HCW_risk_assessment-2020.2-eng.pdf

[20] 20- Greenhalgh T, Wherton J, Shaw S, Morrison C. Video consultations for covid-19. BMJ. 2020;368.m998. doi: 10.1136/bmj.m998 10.1136/bmj.m998 32165352

[21] 21- Machado RA, Souza NL, Oliveira RM, Martelli H Júnior, Bonan PR. Social media and telemedicine for oral diagnosis and counselling in the COVID-19 era. Oral Oncol. 2020;105:104685. doi: 10.1016/j.oraloncology.2020.104685 10.1016/j.oraloncology.2020.104685 PMC7151276 32291154

[22] 22- Daniel SJ, Wu L, Kumar S. Teledentistry: a systematic review of clinical outcomes, utilization and costs. J Dent Hyg. 2013;87(6):345-52. 24357563

[23] 23- Estai M, Kanagasingam Y, Tennant M, Bunt S. A systematic review of the research evidence for the benefits of teledentistry era. J Telemed Telecare. 2018;24(3):147-56. doi: 10.1177/1357633X16689433 10.1177/1357633X16689433 28118778

[24] 24- Alharbi A, Alharbi S, Alqaidi S. Guidelines for dental care provision during the COVID-19 pandemic. Saudi Dent J. 2020;32(4):181-6. doi: 10.1016/j.sdentj.2020.04.001 10.1016/j.sdentj.2020.04.001 PMC7141449 32292260

[25] 25- World Health Organization. Clinical management of severe acute respiratory infection when novel coronavirus (2019-nCoV) infection is suspected [Internet]. Geneva: WHO; 2020 [cited 2020 Apr 19]. Available from: https://apps.who.int/iris/bitstream/handle/10665/331446/WHO-2019-nCoV-clinical-2020.4-eng.pdf

[26] 26- American Dental Association. ADA interim guidance for minimizing risk of COVID-19 transmission [Internet]. Chicago: ADA; 2020 [cited 2020 Apr 21]. Available from: https://www.ada.org/∼/media/CPS/Files/COVID/ADA_COVID_Int_Guidance_Treat_Pts.pdf

[27] 27- American Dental Association. ADA interim guidance for management of emergency and urgent dental care 2020 [Internet]. Chicago: ADA; 2020 [cited 2020 Apr 20]. Available from: https://www.ada.org/∼/media/CPS/Files/COVID/ADA_Int_Guidance_Mgmt_Emerg-Urg_Dental_COVID19?utm_source=adaorg&utm_medium=VanityURL&utm_content=interimguidance-flowcharts&utm_campaign=covid-19

[28] 28- World Health Organization. Considerations for quarantine of individuals in the context of containment for coronavirus disease (COVID-19) [Internet]. Geneva: WHO; 2020 [cited 2020 Apr 21]. Available from: https://apps.who.int/iris/bitstream/handle/10665/331497/WHO-2019-nCoV-IHR_Quarantine-2020.2-eng.pdf

[29] 29- Ather A, Patel B, Ruparel NB, Diogenes A, Hargreaves KM. Coronavirus Disease 19 (COVID-19): implications for clinical dental care. J Endod. 2020;46(5):584-95. doi: 10.1016/j.joen.2020.03.008 10.1016/j.joen.2020.03.008 PMC7270628 32273156

[30] 30- Spagnuolo G, De Vito D, Rengo S, Tatullo M. COVID-19 outbreak: an overview on dentistry. Int J Environ Res Public Health. 2020;17(6):2094. doi: 10.3390/ijerph17062094 10.3390/ijerph17062094 PMC7143628 32235685

[31] 31- Carrouel F, Conte MP, Fisher J, Gonçalves LC, Dussart C, Llodra JC, et al. COVID-19: a recommendation to examine the effect of mouthrinses with β-cyclodextrin combined with citrox in preventing infection and progression. J Clin Med. 2020;9(4):1126. doi: 10.3390/jcm9041126 10.3390/jcm9041126 PMC7230644 32326426

[32] 32- Mukherjee PK, Esper F, Buchheit K, Arters K, Adkins I, Ghannoum MA, et al. Randomized, double-blind, placebo-controlled clinical trial to assess the safety and effectiveness of a novel dual-action oral topical formulation against upper respiratory infections. BMC Infect Dis. 2017;17(1):74. doi: 10.1186/s12879-016-2177-8 10.1186/s12879-016-2177-8 PMC5237564 28088167

[33] 33- Chen C, Zhao B. Makeshift hospitals for COVID-19 patients: where health-care workers and patients need sufficient ventilation for more protection. J Hosp Infect. 2020;105(1):98-99. doi: 10.1016/j.jhin.2020.03.008 10.1016/j.jhin.2020.03.008 PMC7124317 32169615

[34] 34- Cochran MA, Miller CH, Sheldrake MA. The efficacy of the rubber dam as a barrier to the spread of microorganisms during dental treatment. J Am Dent Assoc. 1989;119(1):141-4. doi: 10.14219/jada.archive.1989.0131 10.14219/jada.archive.1989.0131 2760346

[35] 35- Desai BK. Clinical implications of the COVID-19 pandemic on dental education. J Dent Educ. 2020;84(5):512. doi: 10.1002/jdd.12162 10.1002/jdd.12162 PMC7267231 32335909

[36] 36- Emami E. COVID-19: perspective of a dean of dentistry. JDR Clin Trans Res. 2020;5(3):211-3. doi: 10.1177/2380084420929284 10.1177/2380084420929284 32401587

[37] 37- Elangovan S, Mahrous A, Marchini L. Disruptions during a pandemic: gaps identified and lessons learned. J Dent Educ. Forthcoming 2020. doi: 10.1002/jdd.12236 10.1002/jdd.12236 PMC7301023 32500586

[38] 38- Saeed SG, Bain J, Khoo E, Siqueira WL. COVID-19: Finding silver linings for dental education. J Dent Educ. Forthcoming 2020. doi: 10.1002/jdd.12234 10.1002/jdd.12234 PMC7300654 32488877

[39] 39- Prati C, Pelliccioni GA, Sambri V, Chersoni S, Gandolfi MG. COVID-19: its impact on dental schools in Italy, clinical problems in endodontic therapy and general considerations. Int Endod J. 2020;53(5):723-5. doi: 10.1111/iej.13291 10.1111/iej.13291 PMC7262194 32277770

[40] 40- Cagetti MG, Cairoli JL, Senna A, Campus G. COVID-19 outbreak in north italy: an overview on dentistry. a questionnaire survey. Int J Environ Res Public Health. 2020;17(11):3835. doi: 10.3390/ijerph17113835 10.3390/ijerph17113835 PMC7312000 32481672

[41] 41- Bennardo F, Buffone C, Fortunato L, Giudice A. COVID-19 is a challenge for dental education: a commentary. Eur J Dent Educ. Forthcoming 2020. doi: 10.1111/eje.12555 10.1111/eje.12555 PMC7323383 32542796

[42] 42- Meng L, Hua F, Bian Z. Coronavirus Disease 2019 (COVID-19): emerging and future challenges for dental and oral medicine. J Dent Res. 2020;99(5):481-7. doi: 10.1177/0022034520914246 10.1177/0022034520914246 PMC7140973 32162995

[43] 43- Mascitti M, Campisi G. Dental public health landscape: challenges, technological innovation and opportunities in the 21st century and COVID-19 pandemic. Int J Environ Res Public Health. 2020;17(10):3636. doi: 10.3390/ijerph17103636 10.3390/ijerph17103636 PMC7277855 32455777

[44] 44- Elgreatly A, Mahrous A. Enhancing student learning in dental anatomy by using virtual three-dimensional models. J Prosthodont. 2020;29(3):269-71. doi: 10.1111/jopr.13152 10.1111/jopr.13152 32065435

[45] 45- Mahrous A, Schneider GB. Enhancing student learning of removable prosthodontics using the latest advancements in virtual 3D modeling. J Prosthodont. 2019;28(4):471-2. doi: 10.1111/jopr.13044 10.1111/jopr.13044 30838728

[46] 46- Kamate SK, Sharma S, Thakar S, Srivastava D, Sengupta K. Assessing knowledge, attitudes and practices of dental practitioners regarding the COVID-19 pandemic: a multinational study. Dent Med Probl. 2020;57(1):11-7. doi: 10.17219/dmp/119743 10.17219/dmp/119743 32307930

[47] 47- Choi JS, Kim JS. Factors influencing preventive behavior against Middle East Respiratory Syndrome-Coronavirus among nursing students in South Korea. Nurse Educ Today. 2016;40:168-72. doi: 10.1016/j.nedt.2016.03.006 10.1016/j.nedt.2016.03.006 PMC7130744 27125168

[48] 48- Park SW, Jang HW, Choe YH, Lee KS, Ahn YC, Chung MJ, et al. Avoiding student infection during a Middle East respiratory syndrome (MERS) outbreak: a single medical school experience. Korean J Med Educ. 2016;28(2):209-17. doi: 10.3946/kjme.2016.30 10.3946/kjme.2016.30 PMC4951746 27240893

[49] 49- Leng Z, Zhu R, Hou W, Feng Y, Yang Y, Han Q, et al. Transplantation of ACE2- mesenchymal stem cells improves the outcome of patients with COVID-19 pneumonia. Aging Dis. 2020;11(2):216-28. doi: 10.14336/AD.2020.0228 10.14336/AD.2020.0228 PMC7069465 32257537

[50] 50- Ulrich H, Pillat MM. CD147 as a target for COVID-19 treatment: suggested effects of azithromycin and stem cell engagement. Stem Cell Rev Rep. 2020;16(3):434-40. doi: 10.1007/s12015-020-09976-7 10.1007/s12015-020-09976-7 PMC7167302 32307653

[51] 51- Metcalfe SM. Mesenchymal stem cells and management of COVID-19 pneumonia. Med Drug Discov. 2020;5:100019. doi: 10.1016/j.medidd.2020.100019 10.1016/j.medidd.2020.100019 PMC7147223 32296777

[52] 52- Marrazzo P, Paduano F, Palmieri F, Marrelli M, Tatullo M. Highly efficient *in vitro* reparative behaviour of dental pulp stem cells cultured with standardised platelet lysate supplementation. Stem Cells Int. 2016;2016:7230987. doi: 10.1155/2016/7230987 10.1155/2016/7230987 PMC5059612 27774106

[53] 53- Tatullo M, Codispoti B, Pacifici A, Palmieri F, Marrelli M, Pacifici L, et al. Potential use of Human Periapical Cyst-Mesenchymal Stem Cells (hPCy-MSCs) as a novel stem cell source for regenerative medicine applications. Front Cell Dev Biol. 2017;5:103. doi: 10.3389/fcell.2017.00103 10.3389/fcell.2017.00103 PMC5723286 29259970

[54] 54- Barry M, Pearce H, Cross L, Tatullo M, Gaharwar AK. Advances in nanotechnology for the treatment of osteoporosis. Curr Osteoporos Rep. 2016;14(3):87-94. doi: 10.1007/s11914-016-0306-3 10.1007/s11914-016-0306-3 27048473

[55] 55- Jeelani S, Reddy RC, Maheswaran T, Asokan GS, Dany A, Anand B. Theranostics: a treasured tailor for tomorrow. J Pharm Bioallied Sci. 2014;6(Suppl 1):S6-8. doi: 10.4103/0975-7406.137249 10.4103/0975-7406.137249 PMC4157283 25210387

